# Preclinical development of a bispecific TNFα/IL-23 neutralising domain antibody as a novel oral treatment for inflammatory bowel disease

**DOI:** 10.1038/s41598-021-97236-0

**Published:** 2021-09-30

**Authors:** Kevin J. Roberts, Marion F. Cubitt, Timothy M. Carlton, Lurdes Rodrigues-Duarte, Luana Maggiore, Ray Chai, Simon Clare, Katherine Harcourt, Thomas T. MacDonald, Keith P. Ray, Anna Vossenkämper, Michael R. West, J. Scott Crowe

**Affiliations:** 1 VHsquared Ltd., 1 Lower Court, Copley Hill, Cambridge Road, Babraham, Cambridge, CB22 3GN UK; 2 Sorriso Pharmaceuticals, Inc, 12230 El Camino Real, Suite 230, San Diego, CA 92130 USA; 3 Wellcome Sanger Institute, Wellcome Genome Campus, Hinxton, CB10 1SA UK; 4 Blizard Institute, Barts and The London School of Medicine, Queen Mary University of London, 4 Newark St, Whitechapel, London, E1 2AT UK; 5 Present Address: Isogenica Ltd, The Mansion, Chesterford Research Park, Saffron Walden, CB10 1XL UK; 6 Present Address: Institute of Infection, Immunity and Inflammation, University of Glasgow, Sir Graeme Davies Building, 120 University Place, Glasgow, G12 8TA UK; 7 Present Address: Cambridge Institute of Therapeutic Immunology and Infectious Diseases, Jeffrey Cheah Biomedical Centre, Puddicombe Way, Cambridge, CB2 0AW UK

**Keywords:** Antibody fragment therapy, Antibody therapy, Crohn's disease, Ulcerative colitis

## Abstract

Anti-TNFα and anti-IL-23 antibodies are highly effective therapies for Crohn’s disease or ulcerative colitis in a proportion of patients. V56B2 is a novel bispecific domain antibody in which a llama-derived IL-23p19-specific domain antibody, humanised and engineered for intestinal protease resistance, V900, was combined with a previously-described TNFα-specific domain antibody, V565. V56B2 contains a central protease-labile linker to create a single molecule for oral administration. Incubation of V56B2 with trypsin or human faecal supernatant resulted in a complete separation of the V565 and V900 monomers without loss of neutralising potency. Following oral administration of V900 and V565 in mice, high levels of each domain antibody were detected in the faeces, demonstrating stability in the intestinal milieu. In ex vivo cultures of colonic biopsies from IBD patients, treatment with V565 or V900 inhibited tissue phosphoprotein levels and with a combination of the two, inhibition was even greater. These results support further development of V56B2 as an oral therapy for IBD with improved safety and efficacy in a greater proportion of patients as well as greater convenience for patients compared with traditional monoclonal antibody therapies.

## Introduction

The inflammatory bowel diseases (IBD), Crohn’s disease (CD) and ulcerative colitis (UC) are chronic diseases of the gastrointestinal (GI) tract, typically characterized by periods of clinical relapse and remission^1–3^. Current therapies aim to induce and maintain remission, while minimising adverse effects. For IBD patients who fail to respond to standard anti-inflammatory agents, systemic TNFα-neutralising antibodies are currently the most effective treatment^4,5^. However, up to a third of patients do not respond to induction anti-TNFα treatment and in approximately 40% of patients who initially benefit, efficacy is subsequently lost; in the majority of cases due to an anti-drug antibody response^6,7^. The mechanisms underlying the development and chronicity of IBD are multifactorial, and biologics targeting additional proinflammatory cytokines, and pathways for inflammatory cell recruitment, have been developed as alternative therapies for anti-TNFα non-responders^6,8,9^.

Recently, interleukin-23 (IL-23) has been confirmed as another key mediator of mucosal inflammation in both CD and UC^10–14^. IL-23 is a hetero-dimeric cytokine within the IL-12 cytokine family, which also includes IL-12, IL-23, IL-27 and IL-35, and shares a p40 subunit in common with IL-12. IL-12p40 dimerizes with the IL-23p19 subunit to form IL-23, or with an IL-12p35 subunit to form IL-12^15^. IL-23 signals through a heterodimeric IL-23 receptor complex comprising a unique IL-23 receptor (IL-23R) paired with a common IL-12Rβ1 chain that is shared with the IL-12 receptor^16^. Signalling downstream of IL-23/IL-23R/IL-12Rβ1 complex formation occurs via TYK2/JAK2-mediated STAT3 phosphorylation^16^ and subsequent transcription of inflammatory proteins including IL-17^17^. Multiple lines of evidence support a role for the IL-23/IL-23R axis in IBD. IL-23 and IL-23R expression are increased in inflamed IBD mucosal tissue^18,19^ and IL-23 production by macrophages and dendritic cells promotes the expression of further proinflammatory cytokines by mucosal T cells and innate lymphoid cells in the IBD gut^20,21^. Furthermore, the upregulation of mucosal IL-23p19 and IL-23R expression, and the expansion of apoptosis-resistant intestinal TNFR2^+^IL-23R^+^ T cells, are associated with resistance to anti-TNFα therapy in CD patients^22^, leading to the hypothesis that IL-23 antagonists are suitable agents for anti-TNFα-refractory patients. The role of IL-23 in IBD has been clinically validated with both p40- and p19-specific systemic monoclonal antibodies that are efficacious and safe in both CD and UC patients, including those that previously failed anti-TNFα therapy^12–14,23–25^.

In spite of these advances, no current monotherapy is curative in IBD and there is an unmet need for new therapeutics with sustained efficacy across a broader patient population. Combination therapies that target more than one pathway concurrently may be additive or synergistic in terms of efficacy. The safety and potential benefits of combining systemically administered biologic therapies with different therapeutic mechanisms have been investigated in a number of case studies and in a single clinical trial in patients with IBD^26–29^. Results of these studies have shown that in most cases the dual biologic treatments were well tolerated and that efficacy appeared promising for some combinations. However, compared with existing monotherapies, the greater immunosuppressive effects of combining some systemically administered antibodies may result in unacceptable levels of infections and other adverse effects^26^, particularly during long-term treatment. An orally-delivered combination antibody therapy that targets both TNFα and IL-23 and acts locally in the IBD mucosa should circumvent these issues, while providing greater potential for clinical efficacy than either of the monotherapies alone.

Single variable domains of llama heavy chain antibodies (SDAs) retain the potency of conventional antibodies (150 kDa) but have greater intrinsic stability and solubility and are a fraction of the size (12–15 kDa)^30^. SDAs can be engineered for resistance to intestinal proteases^31^ and to reduce the potential for immunogenicity (humanisation). We have developed SDAs for oral administration that have been optimised further for intestinal stability and efficient production in yeast; these we have termed “Vorabodies™”. We have previously reported the preclinical and early clinical development of Vorabody™ V565, a novel protease-resistant TNFα-neutralising SDA of camelid origin, as a treatment for IBD^32–34^. In a recent interventional phase II clinical study in CD patients, improvements in key measures of clinical efficacy were observed after 6 weeks of V565 treatment, with no safety or tolerability issues ( manuscript in preparation). The results of this study have demonstrated the feasibility of delivering a therapeutically effective cytokine-neutralising SDA by oral administration to patients with IBD. Here we describe the selection and characterisation of a novel anti-IL-23 p19-specific SDA that has been fully optimised for oral administration (Vorabody™ V900) and the creation of a bispecific product that combines V900 and V565 into a single molecule (Vorabody™ V56B2) with potent TNFα- and IL-23-neutralising activity. The strategy chosen for coupling the two Vorabodies includes a tryptic-labile peptide linker which is cleaved in the small intestine following oral administration, enabling release of the two monomer arms so that each can bind independently to its target cytokine without interference from the other.

## Materials and methods

### Reagents and antibodies

12G1 and V900 are unoptimised and protease stability optimised anti-IL-23 domain antibodies, respectively. V56B2 is an anti-TNFα/anti-IL-23 bispecific (V565-(G_4_S)_2_-K-(G_4_S)_2_-V900) antibody in which the component V565 and V900 domains have been coupled C to N terminally via a trypsin-sensitive 21mer peptide linker. 12G1 was produced with C-terminal 6xHis and FLAG tags, expressed from *E. coli* and purified using Talon affinity chromatography. The C-terminal tags were not found to affect the potency or protease stability of 12G1. The V900 and V56B2 used in the current study were produced from *S. cerevisiae* or *Pichia pastoris* shake flask cultures and purified using CaptoS ion exchange chromatography. V56B2 was also expressed in a 0.75L methanol-induced bioreactor fermentation from a *Pichia pastoris* mut^S^ PDI strain (Validogen, Graz). ID2A^32^ (SDA isotype control) and V565, protease stable domain antibodies directed against *Clostridium difficile* Toxin A (TcdA) and TNFα, respectively, were produced from *S. cerevisiae* and purified using CaptoS ion exchange chromatography. Biotinylated V900 was generated using an EZ-Link™ Sulfo-NHS-LC-Biotinylation kit (Thermo Scientific, 21435) and the anti-IL-23 activity confirmed by IL-23/IL-23R ELISA. Adalimumab and etanercept were clinical grade from AbbVie and Pfizer, respectively. Biotinylated adalimumab was a custom preparation from LGC, Fordham, UK.

A custom mouse monoclonal antibody specific to V565 (CharT26), with no binding activity for human IgG or V900, was generated at the Wellcome Sanger Institute. Other antibodies and assay reagents were sourced or produced commercially from R&D systems; Recombinant Human IL-23R-FC (#1400-IR-050), Biotinylated polyclonal goat anti-human p40 antibody (BAF219), Recombinant Human IL-12Rβ1 (839-B1-100), Murine IL-23R-Fc (1686-MR-050); Recombinant human gp130-Fc (671-GP), Recombinant mouse IL-17 (421-ML-025), monoclonal mouse anti-h-IFN-y antibody (MAB2852), Recombinant human MMP3 (513-MP-010) and MMP12 (917-MP-010), from Sino Biologicals; Recombinant Human IL-23 (CT012-H08H), Recombinant cynomolgus monkey IL-23R-Fc (90123-C02H), Murine IL-23 (CT028-M08H), Recombinant marmoset IL-23 (CT037-C08H), from Sigma; Goat serum (G9023), Recombinant Human IL-12 (SRP3073), Extravidin-HRP (E2886), Immobilised trypsin agarose (T4019), from Peprotech; Rabbit anti-murine IL-17 biotinylated antibody (500-P265BT), Recombinant human IFN-y (300-02-20), from BioLegend; Recombinant mouse IL-2 (575402), Rat anti-murine-IL-17A monoclonal antibody (506906), from Thermo Fisher; Recombinant soluble human TNFα (PHC3011), from eBioscience; anti-p40 monoclonal capture antibody (14-7129-81), from Genscript; Monorab rabbit anti-camelid single-domain monoclonal antibody (A01860), from Dako; Swine anti-rabbit–HRP (P0217), Rabbit anti-human IgG-HRP specific for gamma-chains (P0214),from Fusion Antibodies; Recombinant cynomolgus monkey IL-23 (Custom preparation), from Invitrogen; Donkey anti-mouse-HRP (SA1-100), from BAC Leiden; Rabbit anti-SDA polyclonal antibody (custom preparation).

### V900 inhibition of cellular responses to IL-23

V900 and 12G1 neutralisation of human IL-23 activity were assessed in a cell-based assay by measuring inhibition of IL-17 production from murine splenocytes that were co-stimulated with murine IL-2 and human IL-23. Cells were cultured in RPMI-1640 medium (Sigma, R0883) supplemented with 10% heat-inactivated foetal bovine serum (Sigma, F7524), 2 mM L-glutamine (Sigma, G7513), 100 U/mL penicillin and streptomycin solution (Sigma P0781), and 10 mM HEPES (Fisher, BPE299-100). Murine IL-17 in the culture supernatant was measured by ELISA. Details of experimental conditions are given in the Supplementary Methods.

### In vitro V56B2 digestion and stability studies

#### Incubation in immobilised trypsin

A full protocol is described in the Supplementary Methods. Briefly, immobilised trypsin agarose was prepared according to the supplier’s instructions and all incubations were conducted in trypsin buffer (1 mM Tris–HCl, 20 mM CaCl_2_, pH 8.0). 1 mg/mL V56B2 in a 25% Trypsin agarose suspension was incubated at 37 °C for 1 h. Immediately after mixing and at selected timepoints during incubation, samples were removed, centrifuged for 2 min at 500 g 4 °C and the supernatants frozen until analysis by SDS-PAGE. The 1 h trypsin-treated V56B2 was dialysed into 1 x PBS containing 0.2 mM PMSF (Sigma 93,482) and analysed by IL-23/IL-23R and biotinylated adalimumab competition ELISAs.

#### Incubation in ex vivo intestinal supernatants

Generation of the mouse small intestinal supernatant (MSIS) and human faecal supernatant (HFS) pools used in this study has been described previously^32^. Antibody solutions were prepared at 250 µg/mL in 1 x PBS, pH 7.4 0.1% BSA, and used in digestion reactions at 20 µg/mL in the relevant matrices. As a 0 h time point, one aliquot was immediately mixed 1:1 with stop solution (2% BSA, 5 mM EDTA, 2 × SigmaFast protease inhibitor cocktail (Sigma S8820), and 1 mM PMSF (Sigma 93,482)) and frozen at − 80 °C. Further aliquots were incubated at 37 °C until the desired time, whereupon an equal volume of stop solution was added and reactions were frozen. Samples were analysed by IL-23/IL-23R ELISA or biotinylated adalimumab competition ELISA for anti-IL-23 or anti-TNFα activity, respectively. Unknown protein concentrations were determined in Graphpad Prism by interpolation against a standard curve.

### ELISA assays

Unless otherwise stated, all antibody standard, test samples and cytokine dilutions were prepared in block buffer (1% BSA in 1 × PBS, pH 7.4). Plates were washed with PBST (1 × PBS, 0.05% Tween 20). For ELISA assays which used IL-23, 4% Marvel milk was also added to the block buffer. Detection antibodies were added in 1% BSA, 1 × PBS. For the analysis of antibodies in MSIS and HFS, or from mouse faecal or intestinal homogenates, 2 × Protease Inhibitor cocktail was also added to the block buffer for all assay steps after plate blocking. NaCl (6 mM) was also added to the block buffer for ELISAs for mouse faeces and intestinal samples for all steps after plate blocking. For the analysis of trypsin-treated V56B2 0.1 mM PMSF was included in the ELISA assay buffer. All ELISA reactions were developed with 100 µL TMB substrate (KPL Microwell Peroxidase substrate System, Seracare 50-76-00), stopped with 50 µL 0.5 M H_2_SO_4_ and plates read at 450 nm OD.

Protocols for all ELISAs are given in the Supplementary Methods.

### Surface plasmon resonance of V900

An anti-p40 monoclonal antibody was coupled to a Biacore CM5 sensor chip then loaded with recombinant human IL-23 at 2 μg/mL or 0.5 μg/mL at 10 µL/min for 60 s. This allowed binding of V900, to the immobilised IL-23p19 subunit, when applied at 0.02, 0.08, 0.3, 1.25 and 5 nM for 300 s on and 300 s off at 30 μL/min.

### SDS-PAGE analyses

Protein samples were loaded into a 10% Bis–Tris NuPAGE gel and electrophoresed in SDS-MES buffer alongside a protein molecular weight standard; EZ-Run™ Prestained Rec Protein Ladder (Fisher BioReagents) or SuperSignal molecular weight protein ladder (Thermo). For protein concentration determination, samples of unknown concentration were loaded at multiple dilutions and electrophoresed alongside a V565 reference standard curve. The gels were imaged in an Imagequant LAS 4000 (Cytiva) using the white transmitted light table and analysed using Imagequant TL software to determine unknown protein concentrations from the V565 standard curve.

#### Inflammatory MMPs

Following activation, recombinant human MMP3 and MMP12 were co-incubated for 19 or 22 h, respectively, with V900 or etanercept using conditions described by Biancheri et al.^35^. Reaction products were analysed by Western blotting as described in the Supplementary Methods.

### In vivo assessment of GI stability and distribution in mice

The care and use of all mice were in accordance with UK Home Office regulations, UK Animals Scientific Procedures Act 1986, under the project licence PPL80/2596. This licence was reviewed and approved by The Wellcome Sanger Institute Animal Welfare and Ethical Review Committee. The mice were housed and fed as described previously^32^. This study is reported in accordance with ARRIVE guidelines. In total, 7 mice were used.

#### GI transit in mice following oral administration

C57BL/6n healthy female mice were pre-dosed by oral gavage with 0.1 mL of a gastro-protective vehicle (0.1 M NaHCO_3_ containing 350 mg/mL Marvel milk), then dosed after 10 min with 0.2 mL 0.1 M NaHCO_3_ containing 90 mg/mL Marvel milk plus either a mixture of 146 µg V900 and 140 µg V565 (4 mice) or, in a separate experiment, a single 245 µg dose of V56B2 (3 mice). The studies were not blinded and all animals were included in the final analyses. All faeces were collected between 0–3 and 3–6 h after dosing and for the V56B2 study the caeca and colons were also removed at 6 h for extraction of the luminal contents. Intestinal luminal contents and faecal samples were weighed, homogenised in 8 volumes extraction buffer (0.6 M NaCl, 1% BSA, 0.05% Tween 20, 2 × SigmaFast protease inhibitor cocktail, Sigma S8820, in 1xPBS) and centrifuged at 13,000 rpm at 10 °C for 20 min. The supernatants were stored at − 80 °C until analysis. Samples were analysed by biotinylated adalimumab ELISA (for V565), IL-23/IL-23R ELISA (for V900) or V56B2 bridging assay. Vorabody levels were determined by interpolation from a plate standard curve and multiplied by the extraction dilution factor to give the concentration in neat faeces or luminal contents.

### Human IBD tissue ex vivo biopsy studies

Research ethics committee approval (reference 10/H0704/73) for studies using human tissue was obtained from the NRES Committee London—City & East. The study was also approved by the local Barts and The London School of Medicine and Dentistry QMUL Joint R&D office. All aspects of the work described were completed following Good Clinical Practice and Good Clinical Laboratory Practice guidelines and were performed in accordance with the relevant guidelines and regulations outlined by the approving institutions. All patients took part in the study after giving informed written consent. Biopsy tissue was obtained from inflamed colonic mucosa during routine endoscopy of patients with UC who had not previously received treatment with anti-TNFα or anti-IL-23 mAbs or ustekinumab. Ex vivo IBD biopsy cultures for the analysis of phosphoproteins were run as described by Nurbhai et al.^34^. UC biopsies were incubated in culture medium for 24 h with the addition of the SDAs, either ID2A (unrelated control SDA), V565, V900 or a mixture of V565 and V900 at the concentrations shown. Tissue samples collected at the end of the experiment were snap-frozen and stored at − 70 °C.

For the analysis of phosphoproteins, tissue samples were thawed, lysed in RIPA Buffer (Sigma-Aldrich, R0278) supplemented with phosphatase inhibitor cocktail 2 (Sigma-Aldrich, P5726) and protease inhibitor cocktail (Sigma-Aldrich, P8340) and the lysate supernatants diluted to 1 mg/mL protein. Lysates (150 µg lysate proteins) were analysed on R&D Systems Proteome Profiler human phosphokinase arrays (ARY003B); a membrane-based antibody array technology for the parallel determination of the relative levels of human protein kinase phosphorylation. Chemiluminescent signals of the arrays were detected on X-ray films, and the spot intensities were measured using ImageJ software. The array signals obtained for the four biopsies in each antibody treatment group were used to calculate average intensity values for each of the 45 phosphoproteins. Values were plotted as histograms or processed using the conditional formatting option in Excel and colours were applied relative to the averaged signal of each phospho-protein in the final array data table. A total phospho-intensity value for each treatment group was calculated by summing the average intensity values of all 45 proteins and results included at the bottom of the array data table.

## Results

### Selection and engineering of anti-IL-23 Vorabody V900

**Figure 1:**
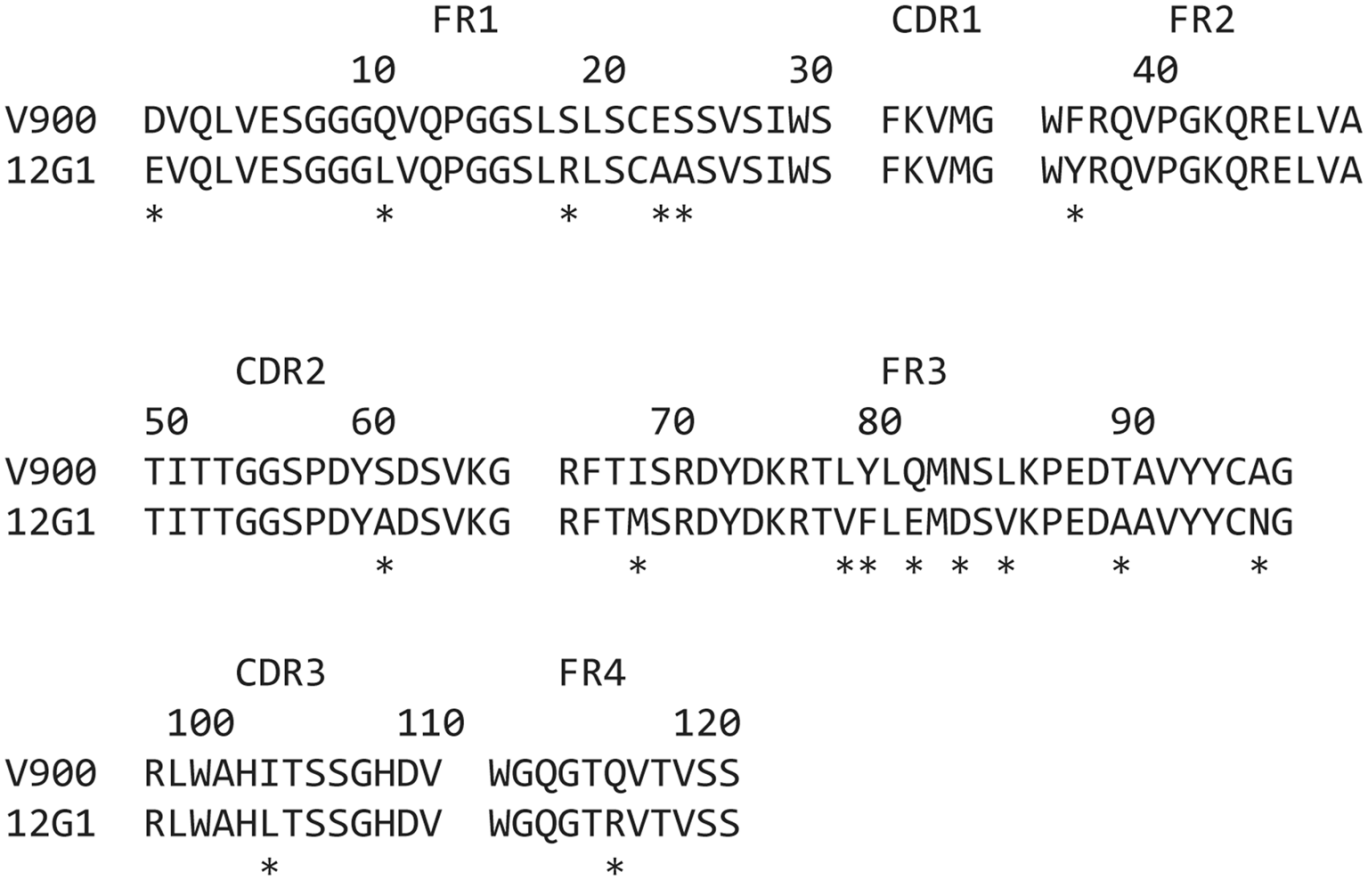
Alignment of the primary amino acid sequences of single domain antibodies 12G1 and V900. Asterisks (*) denote the position of substitutions introduced into the 12G1 sequence to generate Vorabody V900. FR = Framework, CDR = Complementarity determining regions according to KABAT notation.

Llamas were immunised with human IL-23 and boosted periodically using a hyper-immunisation strategy until the antisera contained high titres of IL-23 neutralising antibodies. Peripheral blood lymphocytes from each llama were extracted and used to construct a phage display library from which SDAs with potent IL-23-neutralising activity were isolated. Phage selections were carried out to facilitate enrichment of SDA that bound specifically to the p19 subunit of IL-23. Selected SDAs were subsequently screened by ELISA for their ability to disrupt IL-23 binding to IL-23R, but not IL-23 binding to IL-12Rβ1, or IL-12 binding to IL-12Rβ1. This screen identified five p19-specific SDA sequence families with IC_50_ values in the range 0.2 to 1.5 nM in the IL-23/IL-23R ELISA that had some initial protease resistance. Clones from these families exhibited between 8 and 51% stability after 1 h incubation in mouse small intestinal supernatant (MSIS) and 2.5 to 14.3% stability after 4 h incubation in human faecal supernatant (HFS). Clone 12G1 (Fig. 1 and Table 1) was prioritised as the most potent p19-specific inhibitor of IL-23, with some intrinsic resistance to human intestinal proteases. However, significant engineering was required to improve the protease stability profile to a level suitable for use as an orally delivered IBD therapeutic^32^. This was achieved by substituting amino acid residues from the sequence of SDA 2E9, a highly protease-stable, high yield, SDA that binds to IL-7R^36^ into the corresponding positions in 12G1 (A23E, A24S, Y37F, A60S, E81Q, D83N, V85L, A90T, N96A). Other substitutions improving protease stability (L11Q, R19S, L103I), production in yeast (E1D, R116Q) and further humanisation of the 12G1 sequence (M69I, V78L, F79Y) were also introduced. Clones containing combinations of these substitutions were tested experimentally in multiple iterations for improvements to stability in MSIS and HFS, while retaining the full potency of the parent 12G1 clone. In total, 17 substitutions were introduced into the 12G1 sequence (Fig. 1) to generate V900, a fully optimised Vorabody™ of molecular mass 13.2 kDa, 121 amino acids in length, that was produced in *Pichia pastoris*.

**Table 1:** Activity and protease stability of 12G1 and V900.

Assay	12G1	V900
Human IL-23/IL-23R ELISA	0.2 nM	0.2 nM
Cynomolgus monkey IL-23/IL-23R ELISA	NT	0.7 nM
Cellular potency in mouse splenocytes	9.3 nM	10.7 nM
K_D_	NT	0.032 nM
Mouse small intestinal stability 1	51% (1 h)	51% (4 h)
Mouse small intestinal stability 2	22% (1 h)	40% (4 h)
Human faecal stability	5.6% (4 h)	29% (16 h)

The activity of each SDA was measured in functional receptor/ligand inhibition ELISAs and a primary cell assay using mouse splenocytes. Potency (IC50) and affinity values shown are in nM. Protease stability was measured by incubating SDAs in pooled mouse small intestinal (2 biological replicates) or human faecal supernatants for relevant time periods and detecting residual activity in the human IL-23/23R functional ELISA. Stability values are %, brackets indicate incubation time in hours. NT = not tested.

The IL-23-neutralising activity of V900 was demonstrated using a number of assays. Both 12G1 and V900 potently neutralised human IL-23 binding to IL-23R by ELISA and inhibited the production of IL-17 from murine splenocytes that were co-stimulated with IL-2 and IL-23 (Table 1). These data demonstrated that the amino acid substitutions introduced into 12G1 to create V900 did not attenuate the IL-23 neutralising activity. The binding kinetics of V900 were also analysed by surface plasmon resonance (Biacore) indicating a K_D_ of 32 pM. In species cross-reactivity studies, V900 had no effect on the dose-dependent binding of murine IL-23 to its receptor nor did it bind to marmoset IL-23 captured on marmoset IL-23R. By contrast, V900 fully neutralised binding of IL-23 from cynomolgus monkey (cIL-23) to its cognate receptor, cIL-23R, (Table 1), identifying the cynomolgus monkey as a biologically relevant species for toxicological studies. V900 did not bind to human IL-12, which shares the common p40 subunit with IL-23, or to IL-27, a further member of the IL-12 cytokine family. V900 also showed no interaction with the unrelated cytokines TNFα and IFNγ, confirming the specificity of V900 for IL-23p19 and suggesting any off-target interactions would be extremely unlikely.

### V900 is highly stable in the presence of intestinal proteases

The activities of the major small intestinal proteases, trypsin and chymotrypsin, are conserved across mammalian species, whereas proteases present in the large intestine are produced by epithelial cells, lamina propria mononuclear cells and host species-specific gut microflora^37^. To generate testing conditions that reflect these two environments, 12G1 and the fully optimised V900 were incubated in pooled mouse small intestinal supernatant (MSIS) and pooled human faecal supernatant (HFS) and the activity remaining at each sampling timepoint was measured by IL-23/IL-23R ELISA. 12G1 activity was rapidly degraded in MSIS (22–51% remaining at 1 h) and in HFS (5.6% remaining at 4 h) (Table 1). Engineering markedly improved the protease stability of this SDA, with V900 showing 40–51% activity after 4 h in MSIS and 29% activity after 16 h incubation in HFS.

**Figure 2:**
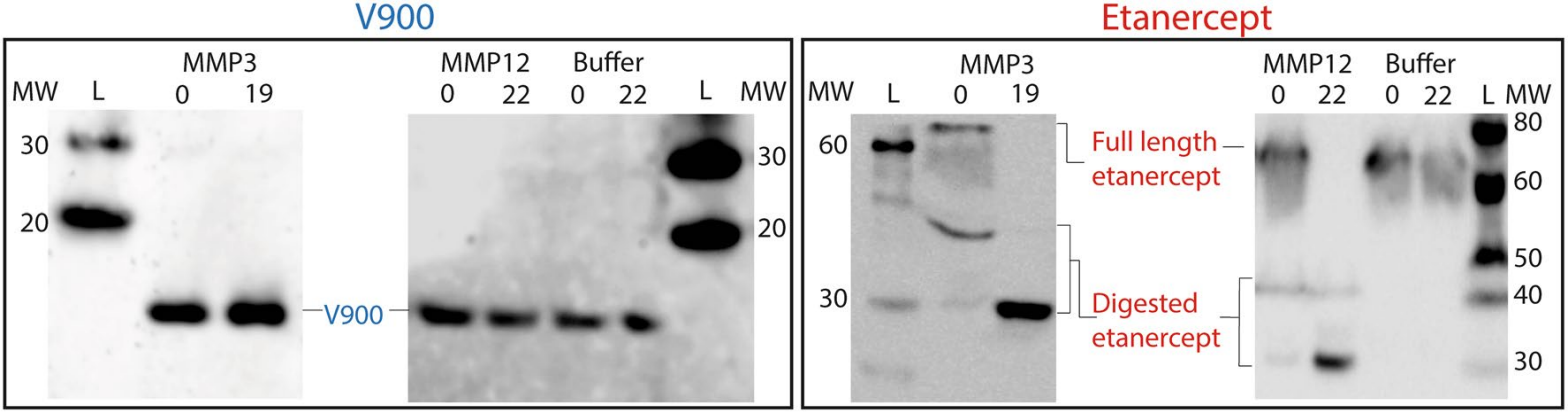
Resistance of V900 to degradation by matrix metalloproteases. V900 and etanercept were incubated with recombinant human matrix metalloproteinases (MMPs) 3 and 12 for 19 or 22 h, respectively. Pre- and post-digestion samples were analysed by Western blotting alongside buffer only (no enzyme) controls. V900 was detected using a polyclonal rabbit α-SDA primary and an HRP-conjugated polyclonal swine anti-rabbit secondary antibody. Etanercept was detected using peroxidase conjugated anti-human IgG specific for Gamma-chains. Due to the high sensitivity of etanercept to MMPs, some degradation was observed in the time zero samples. Blots were visualised using an ImageQuant LAS4000 (Cytiva) on the Chemiluminescensce setting for 1 s (etanercept) or 30 s (V900). L = SuperSignal Prestained ladder. MW = Molecular weight in kDa (vertical numbers). Full length blots are shown in Supplementary Fig. S4.

Levels of inflammatory proteases are elevated in the inflamed mucosa of IBD patients^37^. In particular, activated matrix metalloproteinases (MMPs), such as MMP3 and MMP12 are abundant in these tissues^38,39^. These MMPs exhibit proteolytic activity on a broad range of protein substrates, including native IgG, therapeutic monoclonal antibodies and biologics that contain a human Fc scaffold, such as the TNFα inhibitor etanercept^35^. V900 and etanercept were incubated for 19 h or 22 h with recombinant human MMP3 or MMP12, respectively, and the final products were analysed by Western blotting. Both MMP3 and MMP12 degraded etanercept (Fig. 2). However, neither resulted in degradation of V900 over these time periods (Fig. 2).

### V900 successfully transits the mouse GI tract following oral dosing

**Figure 3:**
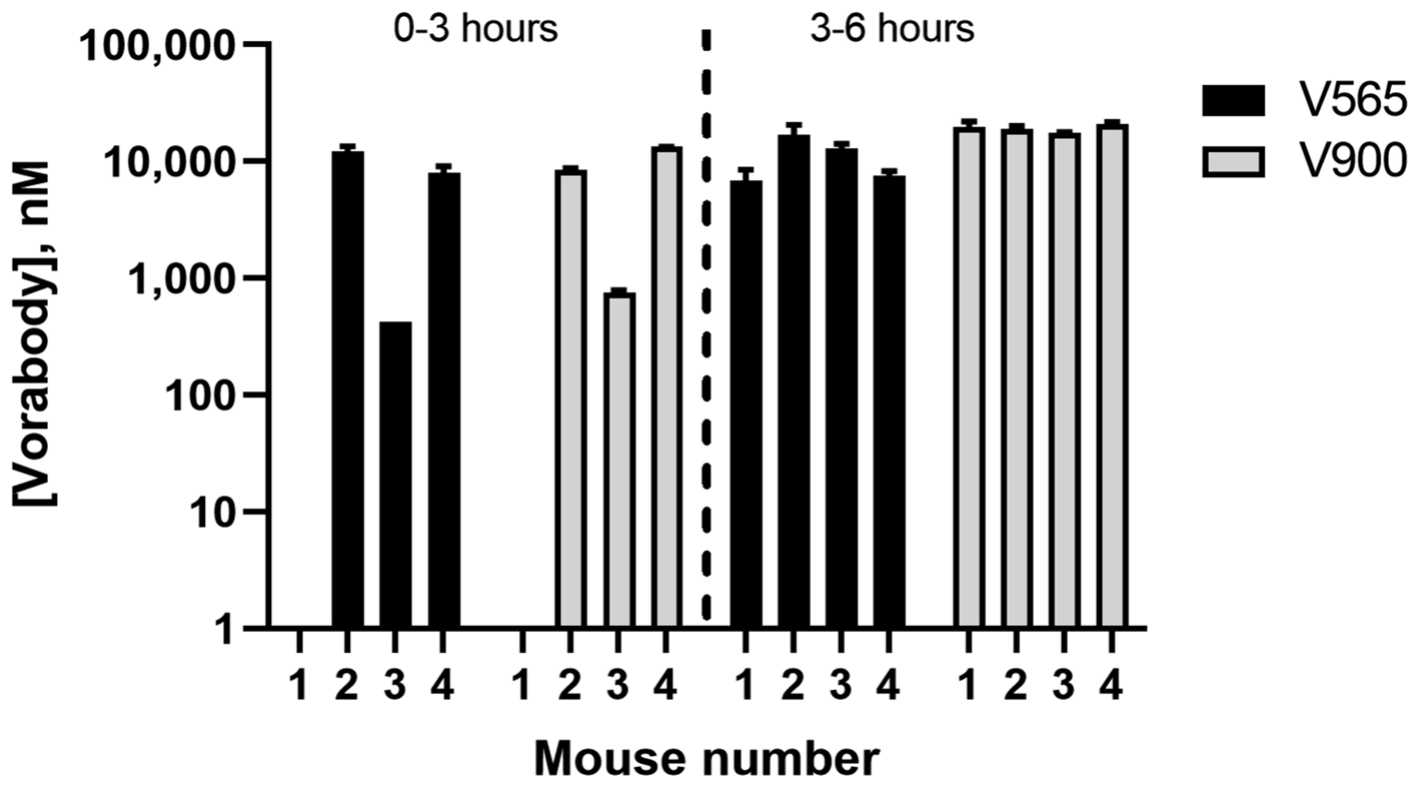
Distribution of V565 and V900 in the faeces of mice following oral administration. Four naïve mice were each administered a mixture of 146 µg V900 and 140 µg V565. Faeces were collected between 0–3 and 3–6 h and V565 and V900 levels in faecal extracts were measured by biotinylated adalimumab competition ELISA and IL-23/IL-23R ELISA, respectively. Concentrations shown are those calculated in the undiluted faeces. Error bars =  +/− SD.

The in vivo stability of V900 during gastrointestinal transit was assessed in mice by oral gavage of V900 together with V565, which had previously demonstrated high stability in the same model as well as in human studies^32^. The V900/V565 mixture was applied in a vehicle containing sodium bicarbonate and milk protein to protect against low pH and degradation in the stomach. Faeces were collected hourly up to either 3 or 6 h and pooled for analysis of TNFα- and IL-23 binding activities by ELISA at these timepoints. Transit of both V565 and V900 through the mouse GI tract was rapid and by 3 h both Vorabodies were detected in the faeces of three out of four mice (Fig. 3). By 6 h, comparable, micromolar concentrations of V565 and V900 were recovered in the stool of all mice, demonstrating the high protease stability of both Vorabodies.

### Formatting of V565 and V900 into the bispecific Vorabody V56B2

The potency, specificity, protease stability and excellent production characteristics of V900 made this SDA a strong candidate for combination with the potent anti-TNFα Vorabody™ V565 (Supplementary Table) to create a single bispecific molecule with dual TNFα- and IL-23-neutralising activities.

The molecular design chosen for this bispecific joined V565 and V900 via a flexible, non-immunogenic (G_4_S)_4_ linker with a central lysine (K) residue to create a deliberate trypsin-cleavage site; (V565-(G_4_S)_2_-K-(G_4_S)_2_-V900). The resulting molecule, V56B2, was expressed from *Pichia pastoris* with yields of 3.2 g/L obtained in a pilot 0.75 L methanol-fed fermentation, and purification direct from the fermentation supernatant by one-step CaptoS ion exchange chromatography.

### V565 and V900 monomers are released rapidly from V56B2 on exposure to trypsin and intestinal fluids

**Figure 4:**
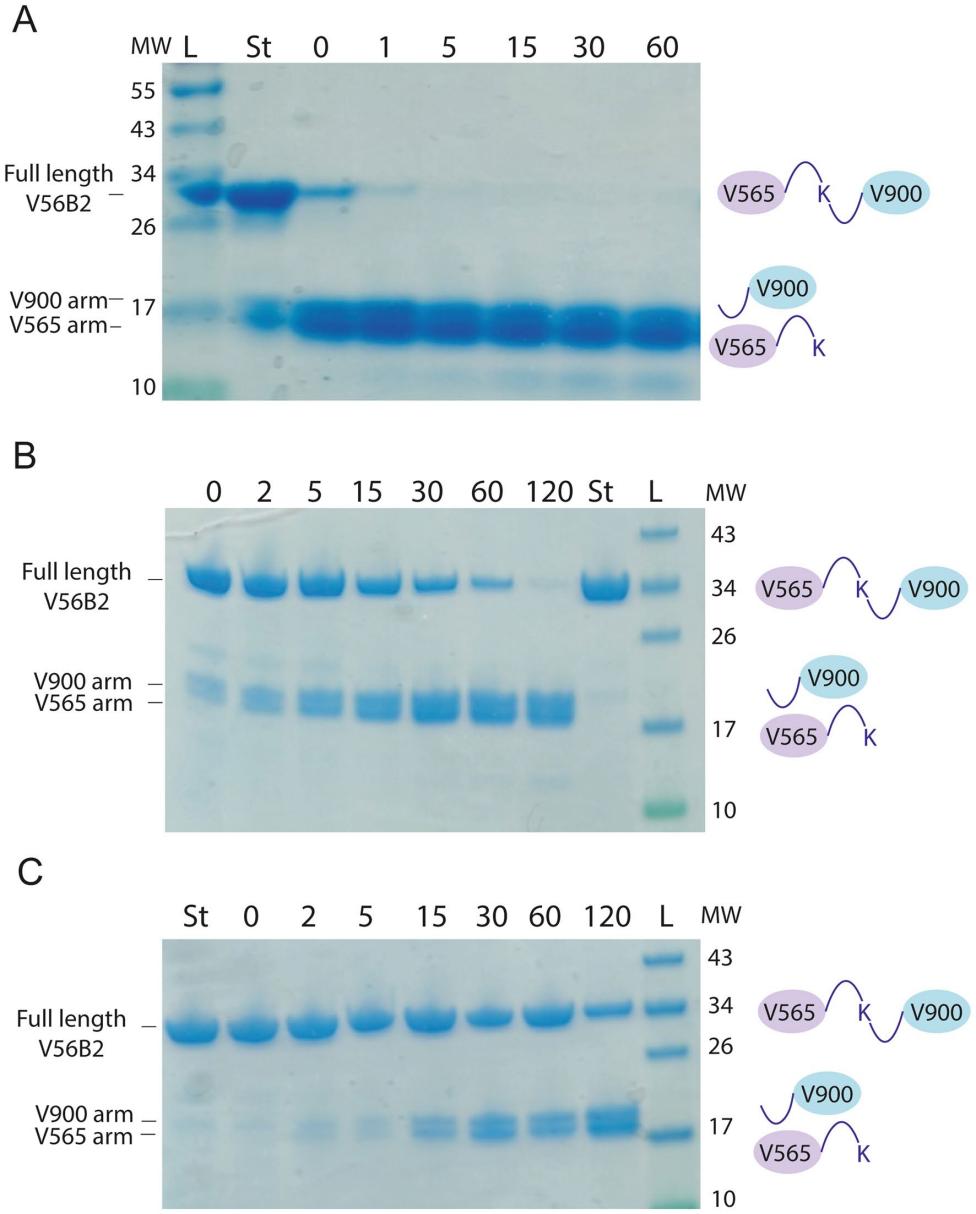
The V56B2 central lysine linker is cleaved by trypsin and in intestinal supernatants. V56B2 was incubated at 37 °C with immobilised trypsin (**A**), 1/1,000 diluted mouse small intestinal supernatant (MSIS) (**B**) or human faecal supernatant (HFS) (**C**). Samples were taken for SDS-PAGE analysis at selected time intervals, shown in minutes (horizontal numbers). Equal volumes were loaded per lane. ‘St’ is undigested V56B2 standard. L = protein standard EZ-Run Prestained Ladder. MW = Molecular weight in kDa (vertical numbers).

Full-length V56B2 was shown to bind to both TNFα and IL-23 simultaneously by ELISA (Supplementary Fig. S1). Dual binding of both cytokines was demonstrated, irrespective of the order of cytokine binding. Incorporation of the trypsin-cleavable linker site in V56B2 will result in monomer separation on exposure to tryptic proteases of the small or large intestine, releasing V565 and V900 for independent binding to their cytokine targets. Rapid separation of the V565 and V900 monomer arms of V56B2 was observed upon incubation with immobilised trypsin at 37 °C (Fig. 4).

Release of the monomer arms was also demonstrated in 1,000-fold diluted MSIS and HFS (Fig. 4B, C). The supernatants were diluted to this extent to allow clear visualisation of V56B2 and the released monomer arms by SDS-PAGE, without interference from supernatant proteins.

### Cleaved V565 and V900 monomers retain full potency

**Figure 5:**
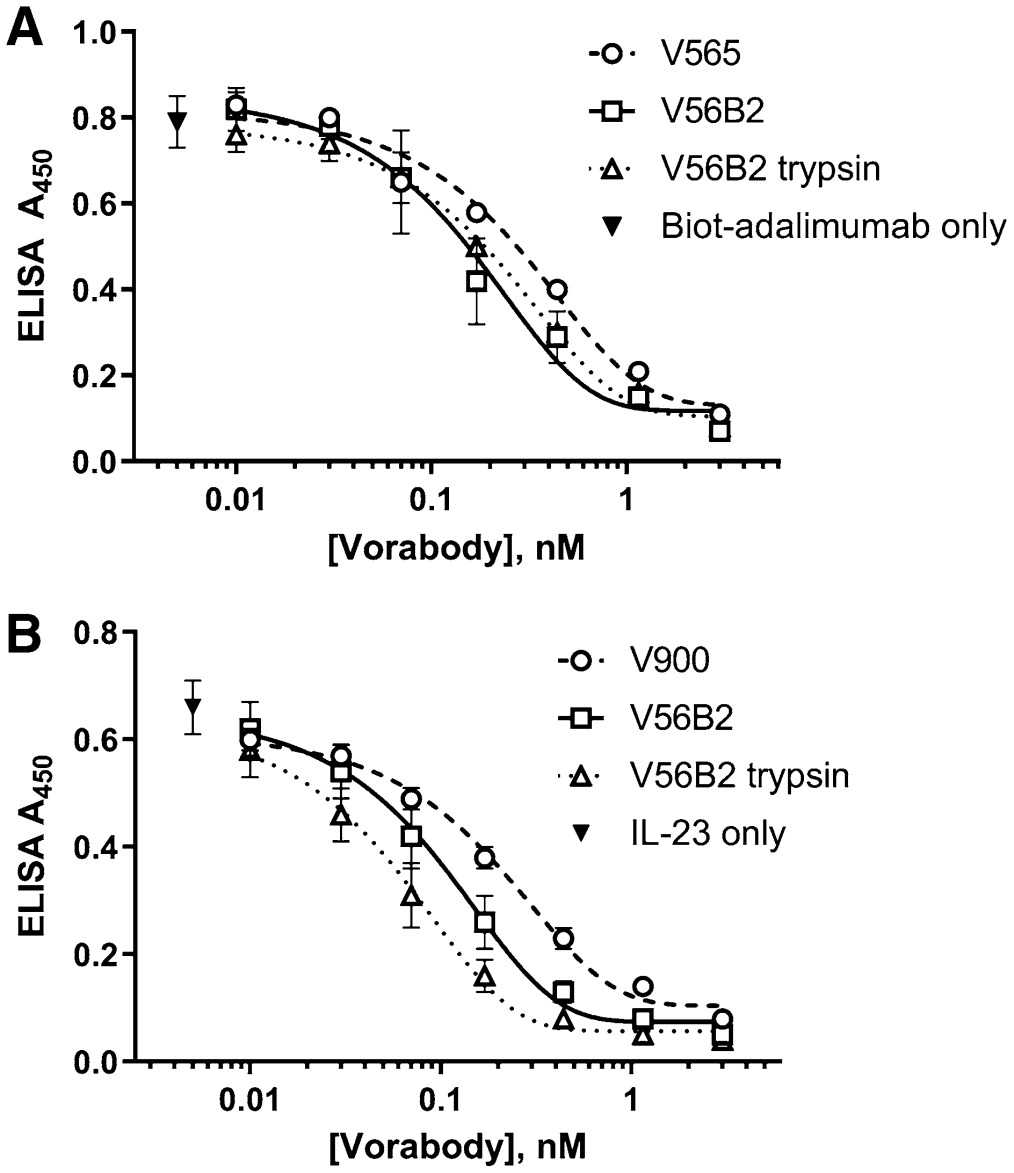
V56B2 retains full anti-TNFα and anti-IL-23 activity. (**A**) V56B2 and the trypsin-liberated V565 monomer arm were tested alongside the V565 parent in the biotinylated adalimumab competition ELISA. Biotinylated adalimumab in the absence of Vorabody was added as a control (**B**) V56B2 and the trypsin-liberated V900 monomer arm were also tested alongside the V900 parent in the IL-23/IL-23R ELISA. IL-23 in the absence of Vorabody was added as an assay control. Error bars =  +/− SD. N = 3.

The liberated V565 and V900 arms from V56B2 were fully resistant to 7.5 units/mL trypsin and demonstrated equivalent activity, compared with the parent V565 and V900 monomers and the untreated, V56B2 parental molecule, after 1 h of trypsin treatment (Fig. 5). In the case of the V900 arm, a marginal increase in potency was observed for the full-length and trypsin-cleaved V56B2 preparation, when compared with the parent V900 monomer (Fig. 5B).

**Figure 6:**
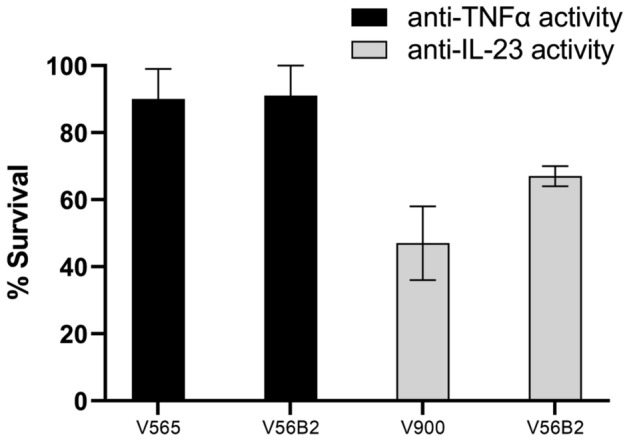
The cleavage products of V56B2 are highly resistant to human faecal proteases. V56B2 and the parent monomers V565 and V900 were incubated in pooled human faecal supernatant for 4 h. This timepoint was selected for accurate observation of differences in stability. Time 0 and 4 h samples were compared for anti-TNFα activity in the biotinylated adalimumab assay (V565 and V56B2) or anti-IL-23 activity in the IL-23/IL-23R ELISA (V900 and V56B2). The remaining activity in each sample at 4 h was calculated as a survival percentage against the 0 h time point. Error bars +/− SD. N = 3.

The liberated arms of V56B2 also retained the stability attributes of V565 and V900 in the neat, pooled human faecal supernatant (Fig. 6), confirming the utility of the molecule for oral delivery and release in the intestinal tract.

### In vivo cleavage and stability of V56B2 following oral administration in mice

To demonstrate the separation of the monomer arms in vivo, 3 mice were dosed with 245 µg V56B2 by oral gavage in the same vehicle described above and faeces were analysed using a bridging ELISA that detects the presence of the full length bihead. Full length V56B2 was detected by ELISA in the gavage solution, but was absent in the faeces even though both monomers were present, demonstrating that the monomer arms were fully liberated from one another during transit through the mouse.

### V565 and V900 inhibit endogenous protein phosphorylation in human IBD tissue

We have previously demonstrated that the TNFα neutralising activity of V565 suppressed the phosphorylation of tyrosine kinase receptors and signalling proteins that are increased in inflamed intestinal tissue samples taken from patients with a diagnosis of IBD^32,34^. A new study was conducted to investigate the effects of combining V565 with the IL-23-neutralising V900 on the levels of phosphoprotein biomarkers in ex vivo cultures of inflamed UC colonic mucosal tissue. To assess the potential for additivity, the antibodies were tested at concentrations that had previously shown close to maximal inhibitory activity in the UC biopsy model (V565 75nM^32^) or in mouse splenocyte cultures (V900 150 nM, inhibition of IL-23 induced IL-17 production, Supplementary Fig. S2), respectively.

**Figure 7:**
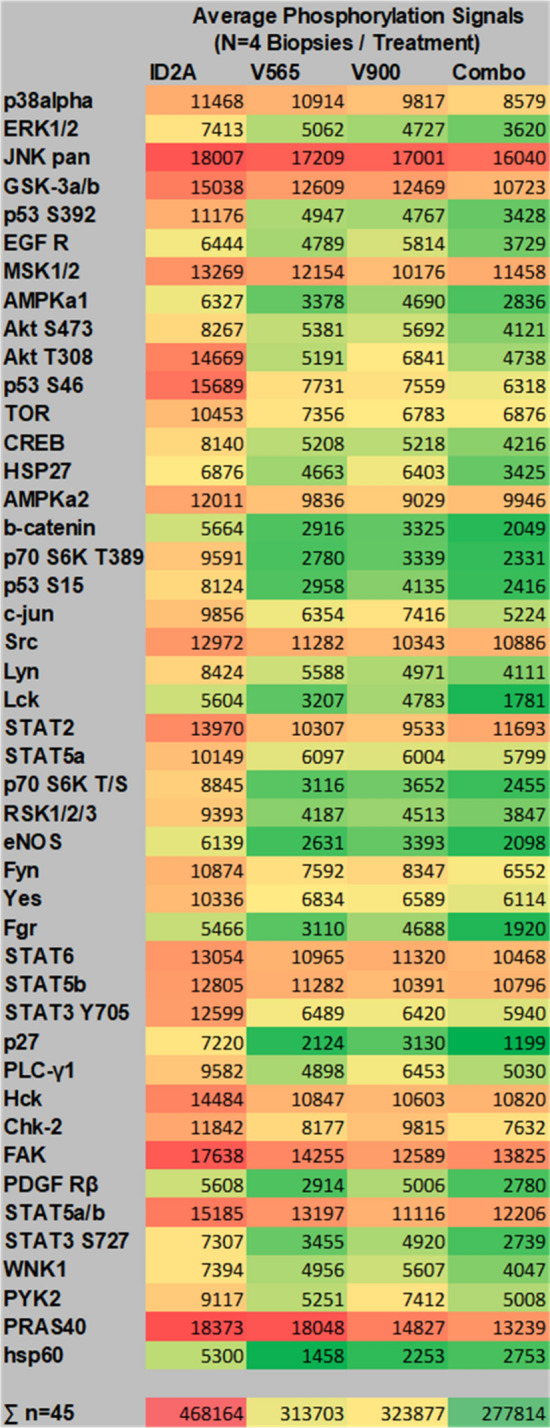
Phospho-array data from four UC patient biopsies grouped according to treatment. Biopsies from four different UC patients were incubated for 24 h with the different single domain antibody treatments (Control (ID-2A) 225 nM; anti-TNFα (V565) 75 nM; anti-IL-23 (V900) 150 nM or V565 75 nM + V900 150 nM combined). Lysates were analysed on R&D proteome profiler human phosphokinase arrays with chemiluminescent detection, image capture on film and quantitation of spot intensities using array analysis software. The array phospho-intensity data were averaged for each treatment (n = 4 biopsies). Values were processed using the conditional formatting option in Excel and colours were applied relative to the averaged signal of each phospho-protein in the final array data set. Red represents proteins with the strongest phosphorylation signals; Green represents proteins with the weakest phosphorylation. The inhibitory effects of the different antibody treatments are demonstrated by a shift from predominantly high levels of phosphorylation (red–orange) for biopsies treated with the isotype control ID2A, to relatively low (yellow to green) phospho-intensity values for biopsies treated with the anti-TNFα or anti-IL-23 antibodies or a combination of the two. For each treatment total phosphorylation values were calculated by summing the averaged (n = 4 biopsies) spot intensities of all 45 analytes.

Following 24 h incubation of colonic biopsies with antibodies ID2A (isotype control), V565, V900 or a mixture of V565 and V900, the phosphorylation levels of a set of tissue proteins were analysed on antibody arrays and average signal intensity values (n = 4 patient biopsies/treatment) calculated for all the 45 phosphoproteins. These results are presented as histograms (Supplementary Fig. S3) or as a heatmap in Fig. 7. The inhibitory effects of the different antibody treatments are demonstrated by a shift from predominantly higher levels of phosphorylation (red–orange) for biopsies treated with the control ID2A, to relatively lower phospho-intensity values (yellow to green) for biopsies treated with the anti-TNFα or anti-IL-23 antibodies or a combination of the two. These results show that when compared with the ID2A control samples, both V565 and V900 each inhibited phosphoprotein levels in the UC biopsy cultures, and that the patterns of inhibition of individual target phosphoproteins on the arrays were broadly similar. Biopsies treated with the combination of antibodies showed greater reductions in the phosphorylation levels of individual analytes relative to those achieved following treatments with the single antibodies V565 or V900, and this trend was reflected in the lower total biopsy phosphorylation levels (∑^n=45^ phosphoproteins).

The combined antibody treatment inhibited phosphorylation levels of a number of proteins on the array by > 50% when compared with the corresponding ID2A treated controls. Of these Lck, Lyn, Fgr, Stat3 and PDGFR are known to have functions involved in the regulation of cells that contribute to inflammation and pathology in IBD, while others have roles in the regulation of endothelial and epithelial cells (eNOS, b-catenin) or have signalling roles in many cell types (Erk 1/2, Akt, AMPKa, p70 S6K, p53, RSK-1). Decreased phosphorylation of these proteins is indicative of decreased cell activation and is consistent with the neutralisation of the endogenously produced proinflammatory cytokines TNFα and IL-23 by V565 and V900.

## Discussion

Systemically administered anti-TNFα antibodies are currently the most effective treatment for patients with moderate-to-severe IBD^4,5^. However, a significant number of patients are either refractory to anti-TNFα treatment or lose response over time^6,7^, leading to the requirement for additional therapies. Clinical validation for the role of IL-23 in IBD has emerged from evaluation of IL-23 neutralising antibodies in patients with CD and UC^40^. Ustekinumab, a monoclonal antibody that inhibits both IL-12 and IL-23 by binding to the common p40 subunit, has been approved for the treatment of moderate to severe, active CD and UC^24,41^. IL-23, rather than IL-12, was subsequently shown to be a key cytokine mediating the development of IBD^42^, and further antibody development has focused on targeting the IL-23p19 subunit. Recently, several IL-23 p19-specific monoclonal antibodies, including brazikumab, risankizumab, guselkumab and mirikizumab have shown efficacy and safety in patients with CD or UC^12–14,25^. Importantly, IL-23 inhibition was effective in both anti-TNFα naïve and anti-TNFα refractory CD patients^12,13^. These data strongly suggest that therapies combining TNFα- and IL-23-neutralising antibodies could achieve improvements in efficacy and/or duration of response in a wider population than either monotherapy alone. This possibility is being investigated in a clinical study to assess the safety and efficacy of combining systemically administered anti-TNFα and anti-IL-23 monoclonal antibodies in patients with UC which is ongoing (NCT03662542).

Administration of anti-TNFα or anti-IL-23 therapies by infusion or subcutaneous injection is inconvenient, can be uncomfortable, and may lead to injection site reactions. Furthermore, a therapy that combines two potent immunosuppressive monoclonal antibodies that are administered parenterally risks increased systemic side effects and may also be limited by the cost of administering two independently manufactured antibody therapies, even accounting for the potential of wider availability of anti-TNFα biosimilars^43^ in the future.

To address these unmet needs, we have developed Vorabody™ V56B2, the first anti-TNFα/anti-IL-23 dual specificity domain antibody for the treatment of IBD. V56B2 combines the benefits of simultaneous inhibition of two key IBD pathways in a single molecule with the convenience and safety of oral drug delivery for local disease-modifying activity and minimal systemic exposure. Production and purification of V56B2 from *Pichia pastoris* is simple, scalable to commercial levels, cost-effective and free of the viral screening requirements associated with mammalian cell-based production systems.

The anti-TNFα arm of V56B2, Vorabody monomer V565, is an SDA that potently neutralises both soluble and membrane forms of human TNFα; V565 is safe in man and has demonstrated efficacy following oral dosing in both UC and CD patients^32,34^ (manuscript in preparation). The anti-IL-23 arm of V56B2, Vorabody V900, is also an SDA and is described here for the first time. V900 potently neutralises the binding of IL-23 to IL-23R and is specific to the p19 subunit of IL-23 with an affinity in the low picomolar range, similar to that described for bivalent anti-p19 monoclonal antibodies that have demonstrated clinical efficacy in IBD^44,45^.

To neutralise cytokines present in the lamina propria (LP) of the inflamed IBD gut, oral therapeutic antibodies must resist degradation by proteases of the intestinal tract and within the inflamed mucosa. In previous studies with V565, stability in ex vivo intestinal supernatants and survival during transit through the mouse intestinal tract was highly predictive of the ability to deliver micromolar V565 concentrations to all regions of the intestinal lumen in humans and non-human primates^33,34^. Here, V900 demonstrated excellent survival in ex vivo mouse small intestinal and human faecal supernatants, comparable with survival levels reported for V565^32^, over time periods that are highly relevant for small and large intestinal transit in man (4 and 16 h, respectively)^46,47^. Moreover, both V565 and V900 were recovered at similar levels in the faeces of mice dosed orally with an equal mixture of the two Vorabodies™ and the favourable protease stability profiles of the monomer arms were fully preserved when formatted into the V56B2 bispecific molecule. Both V565 and V900 are also resistant to matrix metalloproteases MMP3 and MMP12, which can act to cleave therapeutic mAbs and biologics that contain a human Fc region^32,35^. Anti-IL-23p19 SDAs have been described previously that are suitable for systemic administration^48^. However, V900 is the first p19-specific SDA, and V56B2 is the first anti-TNFα/IL-23 bispecific, suitable for oral delivery for the treatment of IBD.

For oral delivery in man, V56B2 will be formulated into gastro-protective enteric-coated minitablets, similar to those used previously to deliver high (micromolar) concentrations of V565 to the mid-small intestine and distal intestine in IBD patients^34^. Upon dissolution of the minitablets in the small intestine, the released V56B2 will be exposed to trypsin which will cleave the central lysine linker, releasing the two monomer arms to bind their respective cytokines independently. Release of V565 and V900 from V56B2 was confirmed here in diluted mouse small intestinal and human faecal supernatants. Accounting for the dilution factor of the supernatant (1,000-fold), separation of the monomer arms from V56B2 would be expected to occur rapidly on release of V56B2 from the minitablets after the enteric coat dissolves, which occurs mainly in the small intestine with some late dissolution in the large intestine^34^. Separation of the monomer arms following oral dosing was also confirmed here in the mouse intestinal transit model.

To test the hypothesis that dual inhibition of TNFα and IL-23 may result in greater efficacy in IBD, ex vivo UC biopsies were incubated with either V565 or V900, a combination of the two, or an isotype control SDA. There were clear reductions in the phosphorylation of a panel of signalling proteins following treatment with V565 and V900, alone and in combination, consistent with the neutralisation of TNFα and IL-23 in the biopsies. In support of the dual therapeutic approach, signalling proteins involved in inflammation and pathology in IBD showed the greatest reduction in phosphorylation with the combination treatment. Despite differences in the signalling of TNFα and IL-23 in IBD, the complexity of the current model and the short treatment period make differentiation of V565 and V900 activity difficult, and the results of the biopsy studies are best interpreted in terms of overall trends^34,49^. For example, IL-23 signalling is mediated, via JAK2, by activated, phosphorylated, STAT3^16^. STAT3 phosphorylation was reduced here by V900 treatment, as expected. However, STAT3 phosphorylation is also upregulated by additional, TNFα-dependent, cytokines including IL-6^50^, which was previously inhibited by V565 in patient biopsies^32^. Indeed, STAT3 phosphorylation was also reduced here by V565 alone. Furthermore, the expression of TNFα was reduced in the ileal and colonic biopsies of CD patients administered intravenously with the anti-IL-23p19 mAb risankizumab^51^. V900 may therefore reduce TNFα-dependent signalling in the current biopsies, independently of V565. Given the complexity of these interactions, it is unsurprising that the greatest reduction in STAT3 phosphorylation was observed in the combination treatment. An important limitation of this ex vivo biopsy culture model is that it does not allow assessment of effects of the antibodies on tissue retention of inflammatory cells or dynamic changes in cell migration thought to be important in inflamed tissue in vivo. Moreover, since the established proinflammatory phenotypes of some important target cells (eg macrophages and T cells) may not be reversible over the 24 h incubation period of the experiment, a longer treatment period may be needed to achieve the full effects of combining V565 and V900. This may be particularly relevant for V900, which may exert an effect on the IL-23-dependent expansion and maintenance of IL-17-producing T-helper Th17 cells^42^. Due to the small number of patient biopsies investigated in this study it was not possible to demonstrate that the differences in phosphorylation levels between V565, V900 or the combination and the control group were statistically significant using a Wilcoxon sign rank test. Nonetheless the patterns of inhibition provided experimental demonstration of the activity of both TNFα and IL-23 neutralising Vorabodies™ in ex vivo disease tissue and is encouraging for the potential efficacy of a bispecific in patients with either TNFα-dependent or TNFα-independent disease.

To achieve simultaneous neutralisation of TNFα and IL-23, the released Vorabody arms of V56B2 must transit from the gut lumen to the lamina propria (LP). Oral dosing of V565 was previously shown to result in reductions in the phosphorylation of protein biomarkers and target engagement with membrane-bound TNFα on cells in the LP of UC patients^34^. Penetration of V565 present in the gut lumen through the disrupted gut lining was also demonstrated in CD patients where V565 was temporarily observed in the serum of the majority of individuals, before being rapidly excreted in the urine (manuscript in preparation). Given the similarities in size of V900 and V565, it is expected that that the trypsin liberated arms of V56B2 will exhibit the same ability to penetrate into the LP, with short-lived systemic exposure. Taken together, the evidence presented here are encouraging towards the desired pharmacokinetic profile for V56B2 as a single product that will deliver saturating, therapeutic concentrations of both V565 and V900 to the inflamed mucosa in IBD patients, for neutralisation of TNFα and IL-23, but without systemic immunosuppression. Given the extensive sequence similarities between camelid SDAs and human variable heavy chain sequences^52^, the further humanisation of V56B2, the tolerogenic oral route of delivery and the short serum half-life of Vorabodies (50 min for V565^33^), immune responses against the separated arms of V56B2 are expected to be minimal, limiting the potential for the secondary loss of response seen with therapeutic mAbs due to development of neutralising anti-drug antibodies (nADAs). Indeed, nADA titres close to baseline were observed after 6 weeks dosing of CD patients with V565 (manuscript in preparation). These nADAs had no detrimental effect on the clinical safety or efficacy of V565 in man and were primarily IgG class antibodies.

Biologic therapies aiming to treat IBD via the oral route have been reported at various stages of development^53–58^. However, V56B2 is unique in that it is the first orally delivered intestinal protease resistant bispecific antibody to provide dual inhibition of two key, clinically validated, IBD pathways without systemic immunosuppression. It has the potential to provide greater clinical efficacy for a longer duration in a larger proportion of IBD patients than biologics targeting single cytokines.

## Supplementary Information

Supplementary Information 1.

## Data Availability

All data generated or analysed during this study are included in this published article (and its Supplementary Information files).
