# Supplementary material for: Preclinical development of a bispecific TNFα/IL-23 neutralising domain antibody as a novel oral treatment for inflammatory bowel disease
Source: Sci Rep. 2021 Sep 30;11:19422. doi: 10.1038/s41598-021-97236-0 (PMC8484351; doi:10.1038/s41598-021-97236-0)
Supplement: Supplementary file 1 — Supplementary Information 1. [file 41598_2021_97236_MOESM1_ESM.docx]

**TITLE: Preclinical development of a bispecific TNFα/IL-23 neutralising domain antibody as a novel oral treatment for Inflammatory Bowel Disease**

**Authors:** Kevin J. Roberts^1,2*^, Marion F. Cubitt^1,5^_,_ Timothy M. Carlton^1^, Lurdes Rodrigues-Duarte^1,5^, Luana Maggiore^1^, Ray Chai^1,6^, Simon Clare^3,7^, Katherine Harcourt^3^, Thomas T. MacDonald^4^, Keith P. Ray^1^, Anna Vossenkämper^4^, Michael R. West^1^ and J. Scott Crowe^1,2^

1. VHsquared Ltd., 1 Lower Court, Copley Hill, Cambridge Road, Babraham, Cambridge, CB22 3GN, UK
2. Sorriso Pharmaceuticals, Inc, 12230 El Camino Real, Suite 230, San Diego, CA, 92130, USA
3. Wellcome Sanger Institute, Wellcome Genome Campus, Hinxton, CB10 1SA, UK
4. Blizard Institute, Barts and the London School of Medicine, Queen Mary University of London, London, 4 Newark St, Whitechapel, London, E1 2AT, UK
5. **Present address**: Isogenica Ltd, The Mansion, Chesterford Research Park, Saffron Walden, CB10 1XL, UK
6. **Present address**: Institute of Infection, Immunity and Inflammation, University of Glasgow, Sir Graeme Davies Building, 120 University Place, Glasgow, G12 8TA, UK
7. **Present address**: Cambridge Institute of Therapeutic Immunology and Infectious Diseases, Jeffrey Cheah Biomedical Centre, Puddicombe Way, Cambridge, CB2 0AW, UK

***Address for Correspondence:** Kevin Roberts, Sorriso Pharmaceuticals, Inc, 12230 El Camino Real, Suite 230, San Diego, CA, 92130, USA.

Email: [KRoberts@Sorrisopharma.com](mailto:Kevin.Roberts@vhsquared.com)

**SUPPLEMENTARY METHODS**

**Mouse Splenocyte Assay**

In this assay, mouse splenocytes were stimulated with a mix of human IL-23 (hIL-23) and murine IL-2 (mIL-2), in the absence or presence of anti-hIL-23 antibodies for 72 hours and culture supernatants were analysed for murine IL-17 concentrations. Splenocyte stimulated IL-17 secretion was inhibited by hIL-23 neutralisation.

Splenocytes isolated from mouse spleen were plated in 96-well round-bottom Corning microplates at 4x 10^5^ cells/50 µL/well in cell culture medium containing 20 ng/mL mIL-2. LPS-stimulated THP-1-cell conditioned culture medium provided the IL-23 for this assay. Antibodies were prepared at 300 nM in the THP-1 cell-derived medium containing 10 ng/mL human IL-23 then diluted in the same culture medium. Fifty microlitres of each antibody dilution mix were transferred into triplicate wells containing 50 µL of cell suspension giving final IL-23 and mIL-2 concentrations of 5 ng/mL and 10 ng/mL respectively. mIL-2 only and mIL-2 + hIL-23 only wells served as negative and positive controls, respectively. Following 3 days incubation at 37^o^C, 5% CO_2_, plates were centrifuged for 2 minutes at 2000 rpm and 50 µL of culture supernatant were recovered from each well. IL-17 concentrations in the culture supernatants were measured using an IL-17 ELISA. IC_50_ values were calculated in Graphpad Prism.

**Immobilised Trypsin Incubation.**

V56B2 was prepared at 2 mg/mL in 1x PBS. Trypsin agarose (Sigma T4019) was prepared according to the supplier’s instructions in trypsin buffer (1 mM Tris-HCl, 20 mM CaCl_2_, pH 8.0), giving a 50% suspension. The 50% trypsin bead suspension was mixed 1:1 with the V56B2 solution. Reactions were prepared on ice and a time zero sample was taken immediately then centrifuged at 4 ^o^C for 2 minutes at 500 g to remove the beads. The remaining reaction volume was incubated for 1h at 37 ^o^C with frequent mixing and 50 µL samples were taken at 1, 5, 15, 30 and 60 minutes. At each time point, 15 µL reaction supernatant was added to 5 µL 4x SDS-PAGE load dye (+DTT) and frozen at – 20 ^o^C. After removal of the 1 hour sample the remaining trypsin-treated V56B2 was recovered by three sequential 2 minute centrifugations and phenylmethane sulfonyl fluoride (PMSF) was then added to a final concentration of 0.2 mM. The solution was dialysed into 1x PBS, 0.2 mM PMSF in a 3.5K MWCO Slide-a-lyzer (Thermo) and then filtered using a 0.22 µM Durapore Ultrafree-MC spin filter (Sigma). Samples were taken for SDS-PAGE analysis and determination of the concentration of the liberated arms. In addition, 10 µL was frozen for analysis in the biotinylated adalimumab and IL-23/IL-23R ELISAs alongside undigested V56B2 and the parent monomer standards.

**ELISA METHODS**

Plate coatings, standards, samples and detection antibodies were added at 50 µL/well. 200 µL of block solution was used for plate blocking. Maxisorb 96 well plates were used in all ELISAs.

**IL-23-IL-23R Neutralisation ELISA**ELISA plates were coated overnight with 50 µL/well 0.3 µg/mL IL-23R-Fc in 5 μg/mL BSA, then blocked with block buffer (4% milk, 1% BSA in 1xPBS). Test antibodies were serially diluted in block buffer and mixed 1:1 with 40 ng/mL recombinant human IL-23 for 30 minutes. Test antibodies mixed with IL-23 were then added to the IL-23R-coated plates. Bound IL-23 was detected with 0.4 µg/mL BAF219 anti-p40 biotinylated polyclonal in 1% BSA containing 0.5% goat serum followed by 1/2,000 Extravidin-HRP in 1% BSA. Unknown V900 or V56B2 sample concentrations were interpolated from the standard curve using GraphPad Prism software.

**Cynomolgus Monkey IL-23-IL-23R Neutralisation ELISA**

ELISA plates were coated overnight with 50 µL/well 0.5 µg/mL cynomolgus
monkey IL-23R-Fc then blocked with 4% milk, 1% BSA. V900 was serially diluted in block buffer, mixed 1:1 with 40 ng/mL recombinant cynomolgus monkey IL-23, then incubated for 30 minutes to allow binding before 50 µL were added to the cIL-23R-coated plates. Bound cIL-23 was detected with 0.4 µg/mL BAF219 anti-p40 pAb containing 0.5% goat serum in 1% BSA, followed by 1/1,000 Extravidin-HRP in 1% BSA.

**Cross Reactivity ELISAs**

To assess the ability of V900 to bind to murine IL-23, ELISA plates were coated overnight with 50 µL/well of 0.5 µg/mL murine IL-23R-Fc in 1 μg/mL BSA then blocked with 4% Milk 1% BSA. V900 was serially diluted in block buffer and mixed 1:1 with 40 ng/mL recombinant murine IL-23 shaking for 30 minutes. 50 µL were then added to the IL-23R-coated plates. Bound IL-23 was detected with 0.4 µg/mL BAF219 anti-p40 biotinylated polyclonal containing 0.5% goat serum in 1% BSA followed by 1/2,000 Extravidin-HRP.

To assess the ability of V900 to bind to marmoset IL-23 or human IL-12, ELISA plates were coated with 50 μL/well 1.25 µg/mL IL-12Rβ1 in 5 μg/mL BSA in 1xPBS overnight at 4°C. Plates were blocked with either 4% milk, 1% BSA for the marmoset IL-23 assay or 1% BSA for the IL-12 assays. Two-fold dilution series of each cytokine with a top concentration of 100 ng/mL were prepared in the relevant block buffers and 50 μL were added to the plate. Each cytokine ELISA plate was then incubated with 50 nM biotinylated V900 and, in a separate set of wells, 50 nM biotinylated anti-p40 pAb (BAF219) in 1% BSA, followed by 1/1,000 Extravidin-HRP for detection. A signal in the assay indicated that the antibody or ICVD had bound to the cytokine tethered to the plate via the p40-IL-12Rβ1 interactions.

To assess the ability of V900 to bind to human IFN-y, Maxisorp 96-well plates were coated overnight with 1 μg/mL monoclonal mouse anti-h-IFN-y antibody (R&D systems, MAB2852) in 5 μg/mL BSA. A two-fold dilution series of hIFN-y was made in 1% BSA, beginning at 100 ng/mL, and added to the ELISA plate for 2 hours. The captured dilution series were incubated with 50 µL 50 nM biotinylated V900 or 1 μg/mL Biotinylated-rabbit anti-IFN-y (Peprotech, 500-P32BT), followed by Extravidin-HRP at 1/1,000.

To assess the ability of V900 to bind to IL-27, an ELISA was conducted using the RnD Systems DuoSet IL-27 ELISA kit (DY2526) according to manufacturer’s instructions. Briefly, the ELISA plate was coated with an anti-IL-27 capture antibody and blocked. IL-27 was then applied to the plate. Serial dilutions of either biotinylated V900, or the positive control biotinylated anti-IL-27 antibody, were then applied to the plate, followed by streptavidin-HRP.

**Biotinylated Adalimumab Competition ELISA**

This ELISA measures competition between antibodies and biotinylated adalimumab for the same epitope on TNFα, which is bound to the ELISA plate. The assay signal is therefore reduced in the presence of increasing [V565]. Plates were coated overnight with 50 µL/well 100 ng/mL TNFα in 100 µg/mL BSA. Antibody dilution series were made at 2x the final assay concentration, mixed 1:1 with 0.6 nM biotinylated adalimumab and added to the assay plates, followed by 1/3,000 Extravidin-HRP. Biotinylated adalimumab only controls were included on each plate. Unknown V565 or V56B2 sample concentrations were interpolated from the standard curve using GraphPad Prism software.

**V56B2 Bridging ELISA**

To detect full-length V56B2, murine mAb CharT26 is a high affinity antibody for V565, but does not recognize human IgG, or V900. By contrast, the Genscript rabbit anti-SDA mAb recognizes V900, but not V565. ELISA plates were coated overnight with 50 µL/well 0.75 µg/mL GenScript anti-SDA mAb then blocked with block buffer (1% BSA in 1x PBS). V56B2 was serially diluted in block buffer and added to the ELISA plates, followed by 0.5 µg/mL CharT26 and then 1/5,000 donkey anti-Mouse-HRP, both in 1% BSA. The bridging ELISA is able to detect full-length V56B2 in the presence of intestinal and faecal material.

**Dual TNFα and IL-23 Binding ELISA**

ELISA plates were coated overnight with 50 µL/well 250 ng/mL TNFα in 100 µg/mL BSA 1 x PBS then blocked with blocked buffer (1 % BSA, 4 % Marvel in 1x PBS). V56B2 was serially diluted in block buffer and added to the ELISA plates, followed by 40 ng/mL IL-23 in block buffer, 0.4 µg/mL biotinylated anti-p40 (BAF219) in 1% BSA and 1/2,000 Extravidin-HRP in 1% BSA. An alternative version of the assay was also performed in which the V56B2 and IL-23 were made up at double the final assay concentrations, mixed 1:1 and pre-incubated for 1.5 hours before addition to the plate. Detection with BAF219, Extravidin-HRP were conducted as described above.

**Mouse IL-17 Detection ELISA**

ELISA plates were coated overnight with 50 µL/well 1 µg/mL rat anti-mouse IL-17 then blocked with 1% BSA. Recombinant mouse IL-17 (mIL-17) was serially diluted (0.041 ng/mL to 10 ng/mL) in 1% BSA to generate a standard. 50 µL of the standard and culture supernatants recovered from the mouse splenocyte assay were added to the anti-mIL-17 coated plates. Bound mIL-17 was detected with 0.25 µg/mL Biotinylated Rabbit polyclonal anti-mouse IL-17, followed by 1/2,000 Extravidin-HRP.

**Western Blotting Analysis of MMP Digest Samples**

Samples were diluted to the equivalent of 11.25 ng/µL test compound in load dye and 15 µL was loaded (170 ng of each test compound/lane) into a 10% Bis-Tris NuPAGEgel (ThermoFisher, NP0316BOX). The equivalent volume of the ‘no test compound’ controls was also loaded. Super Signal MW protein Ladder (ThermoFisher, #84785) was added as a standard at 5 µL/lane. Samples were electrophoresed in SDS-MES buffer and transferred to nitrocellulose membranes (ThermoFisher, IB3010) using an iblot (ThermoFisher, 7 minute semi-dry transfer program 3). The membranes were blocked overnight at 4 ^o^C in block solution (1% BSA, 2 % Marvel, 0.05% Tween20, 1xPBS pH7.4). V900 was detected using primary: polyclonal rabbit α-SDA at 1/1,000 in block solution and Secondary: HRP-conjugated polyclonal swine anti-rabbit at 1/1,000 in block solution. Etanercept was detected using peroxidase conjugated anti-human IgG specific for Gamma-chains at 1/1,000 in block solution. Blots were washed for 6 x 5 minutes in 25 mL PBST (1xPBS, 0.1% Tween20) between each incubation step to remove non-specifically bound antibody. SuperSignal™ West Pico Plus Chemiluminescent Substrate (Thermo, 34087) was used to develop the blots, which were visualised using an ImageQuant LAS4000 (Cytiva) on the Chemiluminescensce setting, High Precision, 30 seconds exposure for V900, 1 second exposure for etanercept.

**SUPPLEMENTARY DATA**

**TABLES**

| Property | Assay Description | V565 | V900 |
| --- | --- | --- | --- |
| Molecular Weight kDa | *In silico* | 12.6 | 13.2 |
| Antigen binding affinity K_D_ (pM) | SwitchSense measurements (V565), Biacore SPR (V900) | 17 | 32 |
| Potency – ELISA  (IC50, nM) | TNFR2-sTNFα Interference (V565), Inhibition of IL-23 binding to IL-23R (V900) | 0.4 | 0.2 |

Properties of V565 and V900.

**SUPPLEMENTARY FIGURES**


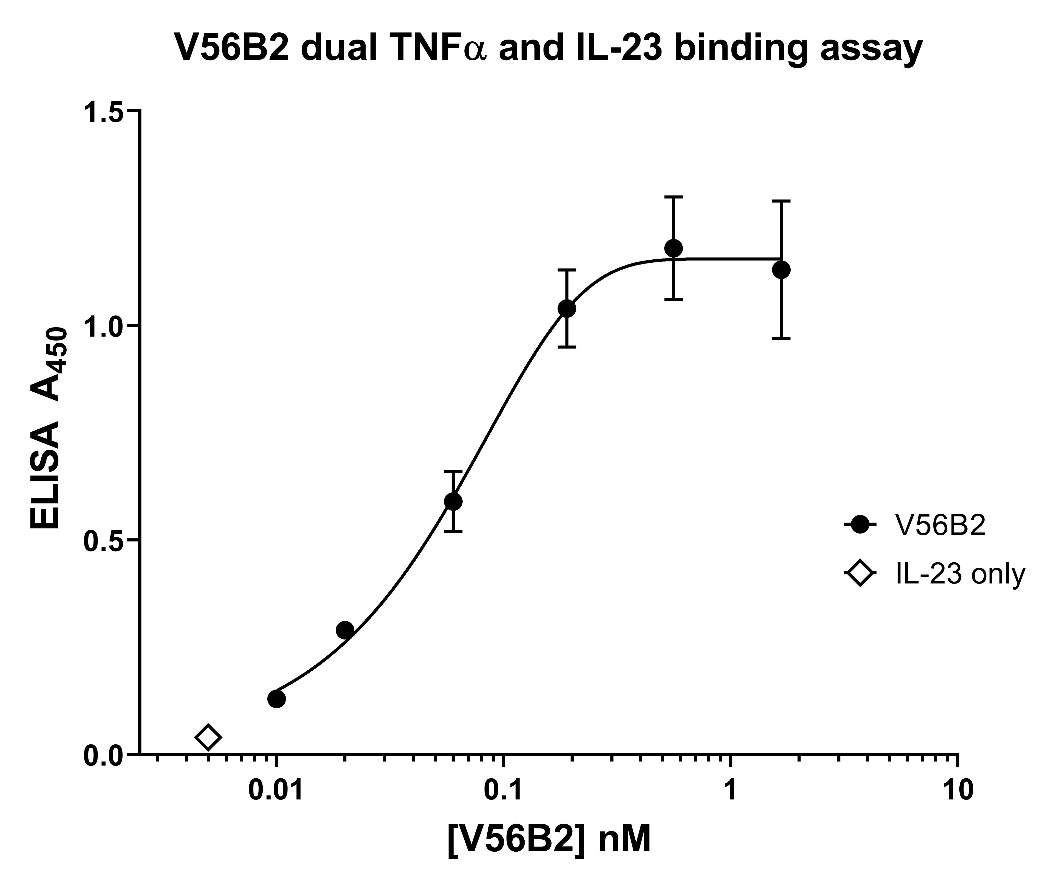


**S1. V56B2 can bind to TNFα and IL-23 simultaneously.** ELISA plates were coated with TNFα and incubated with a dilution series of V56B2, followed by IL-23. Detection of IL-23 by a polyclonal anti-P40 was observed only in the presence of V56B2. In the absence of V56B2, IL-23 was not captured in the assay (IL-23 only). Error bars=+/−SD. N=3.


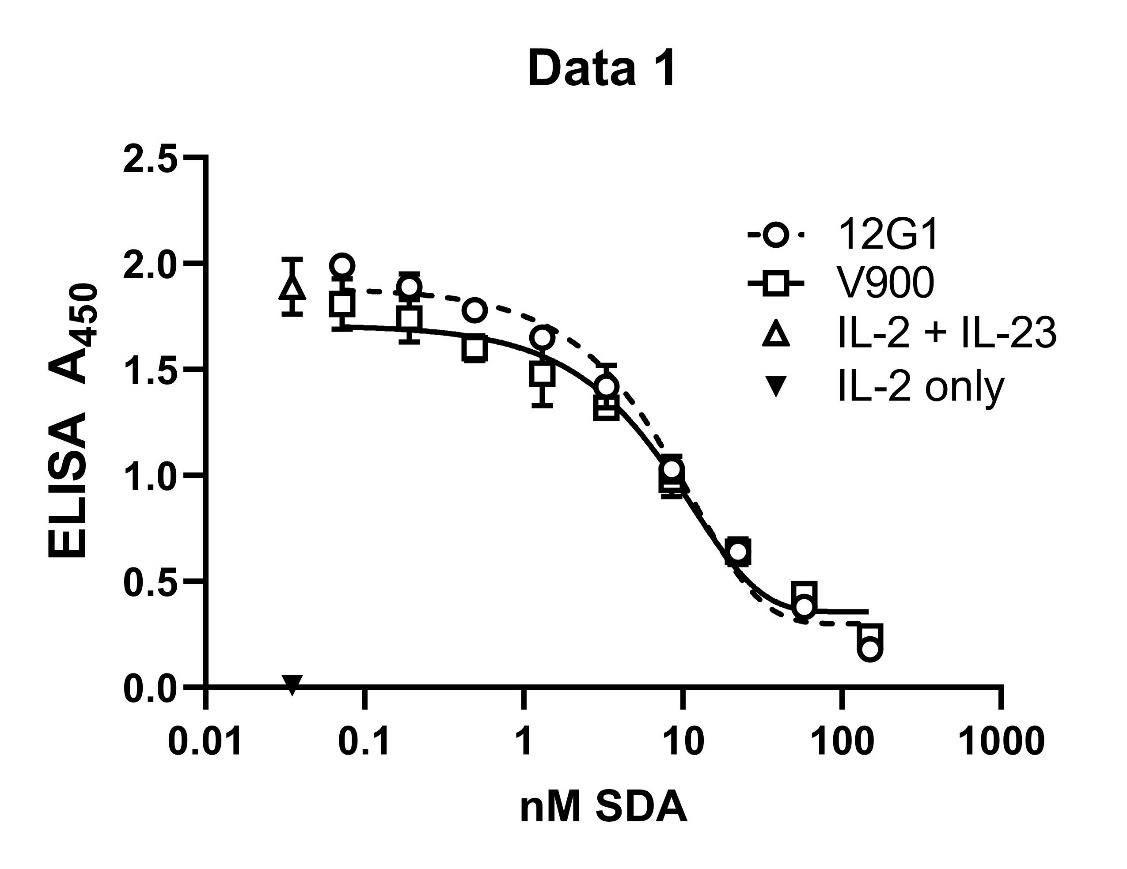


**S2. V900 and 12G1 inhibit IL-23-induced IL-17 secretion from murine splenocytes.** Murine splenocytes were stimulated with a mixture of human IL-23 and murine IL-2 in the presence of the single domain antibodies (SDAs) V900 or 12G1. IL-17 in the supernatant was measured by mouse IL-17 detection ELISA. IL-23 plus IL-2 and IL-2 only treatments were added as controls. Error bars=+/−SD. N=3.


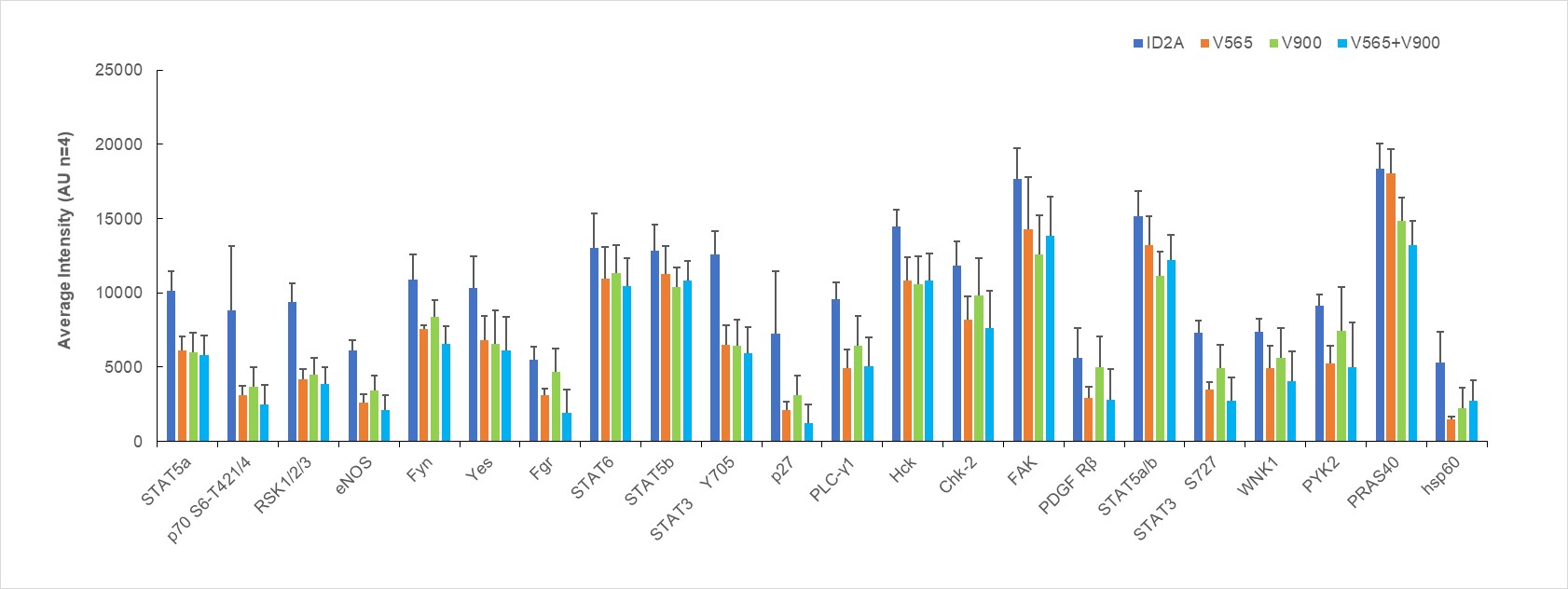

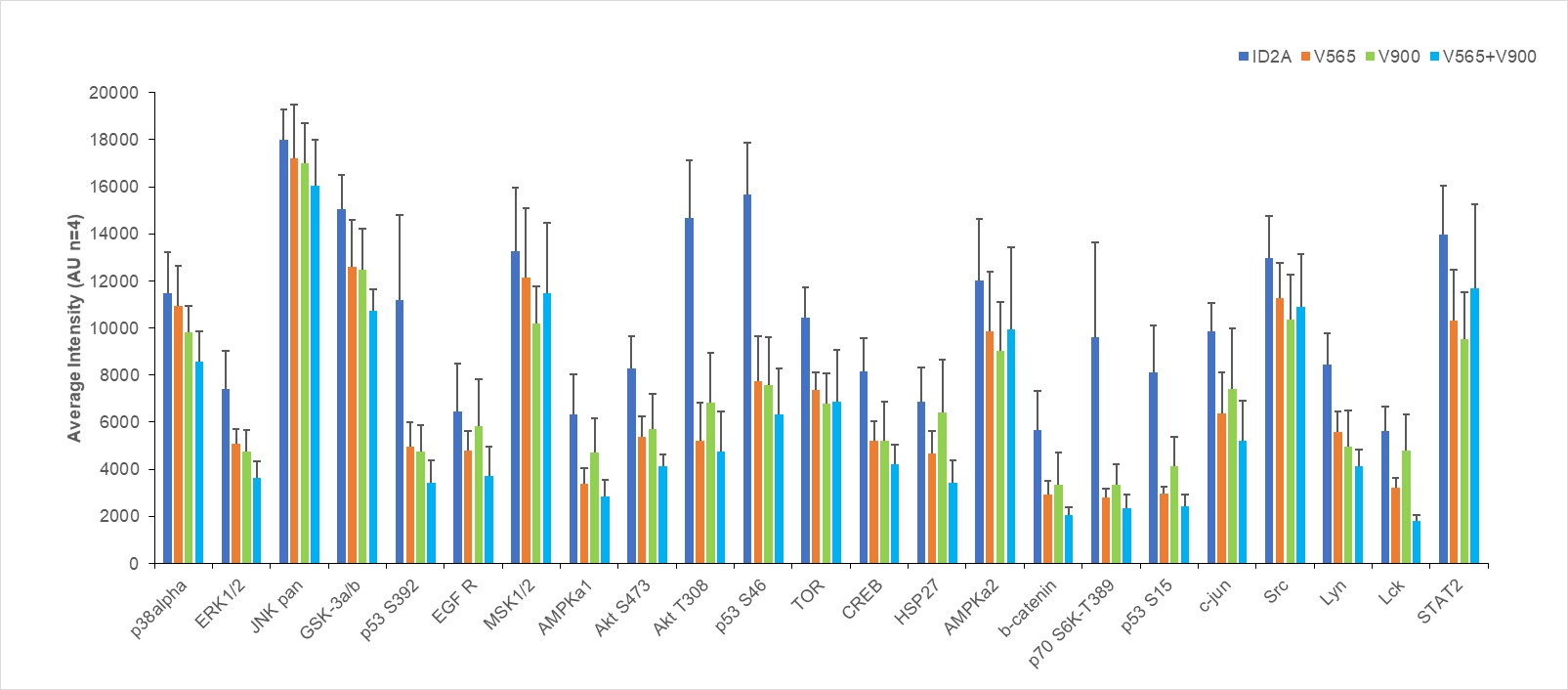


**S3 Phospho-array data from four UC patient biopsies grouped according to treatment.**

Biopsies from four different UC patients were incubated for 24h with the different single domain antibody treatments (Control (ID-2A) 225 nM; anti-TNFα (V565) 75 nM; anti-IL-23 (V900) 150 nM; V565 75 nM + V900 150 nM combined). Lysates were analysed on R&D proteome profiler human phosphokinase arrays with chemiluminescent detection, image capture on film and quantitation of spot intensities using array analysis software. The array phospho-intensity data were averaged for each treatment (n=4 biopsies) and intensities obtained for all 45 proteins analysed on the array are shown. Error bars = standard errors of the mean.


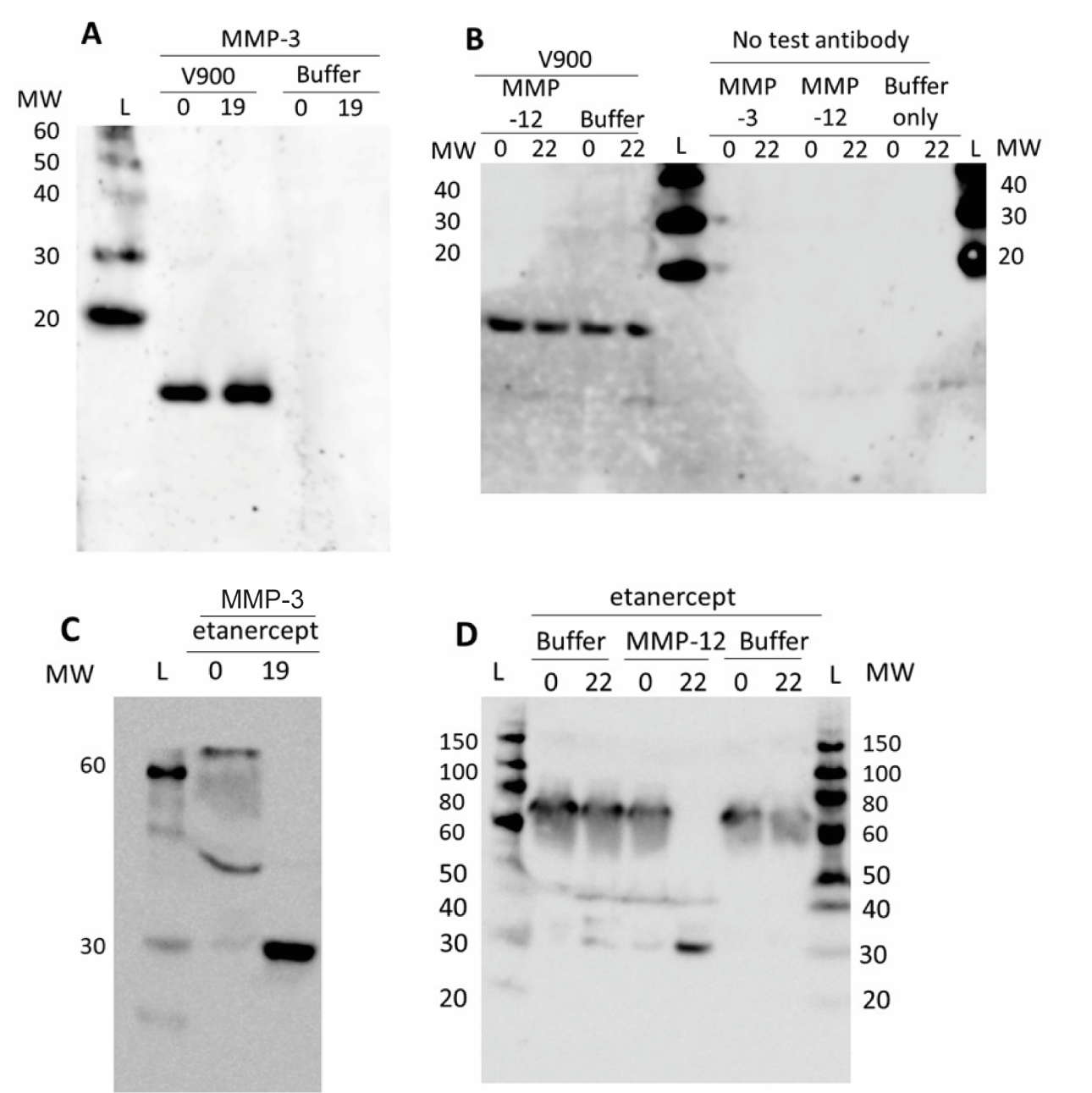


**S4 Resistance of V900 to degradation by matrix metalloproteases.** Full length images from 4 separate blots (A-D) are shown. V900 (A and B) and etanercept (C and D) were incubated with recombinant human matrix metalloproteinases (MMPs) 3 and 12 for 19 or 22 hours, respectively. Pre- and post-digestion samples were analysed by Western blotting alongside buffer only (no enzyme) controls. V900 was detected using a polyclonal rabbit α-SDA primary and an HRP-conjugated polyclonal swine anti-rabbit secondary antibody. Etanercept was detected using peroxidase conjugated anti-human IgG specific for Gamma-chains. Blots were visualised using an ImageQuant LAS4000 (Cytiva) on the Chemiluminescensce setting for 1 second (etanercept) or 30 seconds (V900). L = SuperSignal Prestained ladder. MW = Molecular weight in kDa (vertical numbers). Following transfer, blots were trimmed to the sizes shown above prior to the addition of detection antibodies and exposure.
